# Determination of non-metallic inclusions in a continuous casting slab of ultra-low carbon interstitial free steel by applying of metallographic method, electrolytic method and RTO technique

**DOI:** 10.1038/s41598-018-36766-6

**Published:** 2019-02-27

**Authors:** Jing Guo, Shu-sen Cheng, Hanjie Guo, Yaguang Mei

**Affiliations:** 1 Metallurgical and ecological engineering, University of Science and Technology Beijing, Beijing, 100083 P.R. China; 2 Key Laboratory of Special Melting and Reparation of High-end Metal Materials, Beijing, 100083 P.R. China

## Abstract

Metallographic, electrolytic method and RTO(room temperature organic) technique were applied in the present study to more accurately determine non-metallic inclusion in a ultra-low carbon interstitial free(IF) steel and further to confirm their origination in a Compact Strip Production Process (CSP Process) continuous casting (CC) slab. Results show that inclusions detected by metallographic method usually appear relative smaller size and are mainly Al_2_O_3_ based or TiN based in composition, whereas those extracted by electrolytic method usually have larger size and are much more calcium-silicate based in composition. In addition, inner structures of extracted inclusions were detected by applying of RTO technique. The large size calcium-silicate based inclusions are confirmed high possibilities originating from mold flux and/or tundish flux entrapment, which are less affected by the liquid steel composition; while the smaller ones are generally endogenous inclusion precipitating during the refining or solidification process that strongly depending on the liquid steel composition and temperature.

## Introduction

The surface quality problem of hot and/or cold rolled strip is always one of the most concerns since it is relative to the steel quality and price directly. The surface quality of rolled strips is influenced by each operation of the foregoing process, including refining, continuous casting, reheating, hot rolling, pickling and cold rolling,. Many researchers^1–3^ have paid many efforts to explain the mechanism to cause defects in cold rolled trip surface and most researchers agreed with that the non-metallic inclusion was one of the major causes to generate the surface cracks in a rolled strip.

**Figure 1:**
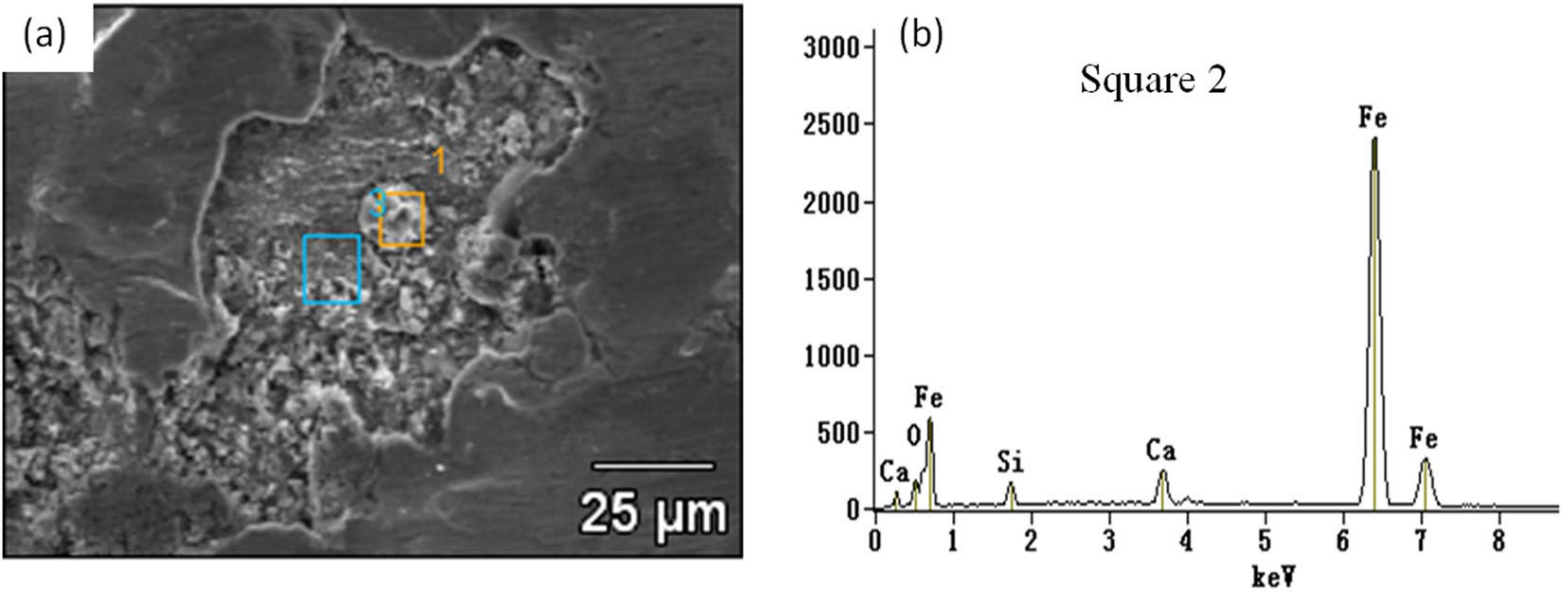
Calcium-Silicate based inclusion in a sliver crack appeared on a cold strip(**a**) SEM photo, and (**b**) EDS diagram).

The TSCR(Thin-slab Casting and Rolling) process is with high casting speed thus the fluctuation of meniscus in the mold is much more volatile and steel cleanness is more difficult to control. Therefore, there are more negative effects of inclusion on surface quality for the rolled strip. In fact, the relative poor trip surface quality is one of the biggest challenge to produce the ultra low carbon Interstitial free(IF) steel by applying TSCR process. Many researchers^4–8^ attempted to promote the strip surface quality by modifying inclusion composition and morphology based on thermodynamic calculation. Two of the authors’ previous works^7,8^ have tried to modify the Al_2_O_3_ based inclusion into liquid ones to improve their deformability during CSP rolling process. But it seems still not enough to solve the surface quality problem completely. For example, a number of SiO_2_-CaO based inclusions were frequently detected in some strip cracks, as shown in Fig. 1. This is very difficult to be explained by current non-metallic inclusion formation theory since very low silicon content in IF steel(usually lower than 0.02%).

In this manuscript, two technology process, with Ca-treatment and without Ca-treatment respectively, to produce ultra-low carbon interstitial free(IF) steel were carried out in CSP Process. Moreover, inclusions in a casting slab were detected by applying metallographic,electrolytic method and RTO technique in the present study in order to obtain more information of inclusions in a slab and to further confirm the origin of the non-metallic inclusions, in particular the CaO-SiO_2_ based ones.

## Experimental and Research Methods

The present trials were carried out in a 120 ton ladle of a steel plant in China. Two kinds of routes listed in Table 1 were performed in order to confirm influence of different liquid steel and slag composition on the behaviors of inclusion in a slab. In the first route, Al-Fe alloys were firstly added into liquid steel for strong preliminary deoxidation during basic oxygen furnace (BOF) tapping. Thereafter, high basicity slag was used in ladle furnace(LF) for desulfurization. After that, the ladle was transferred to Ruhrstahl-Hereaeus(RH) station for further decarbonization and degassing. Thereafter, approximately 200 meters calcium wire was injected for inclusion modification. A 30 ton tundish and an approximately 58 mm in thickness funnel type mould are equipped in the caster. The biggest differences of the second route from the first one were that ladle after BOF tapping was shifted to RH station directly and no Ca treatment were conducted after Al deoxidation and titatiun alloying in RH refining. Tables 2 and 3 show the average composition of liquid steel and slag of three heats in one cast at the end of RH refining process, respectively. The composition of steel samples were analyzed by ICP-AES(Inductively Coupled Plasma - Atomic Emission Spectrometry) method as well as Carbon/Sulfur analyzer. The composition of the slag was analyzed by an X-ray fluorescence spectrometer. In the present trials, steel samples were taken at three positions in a casting slab along the wide face central line: at center (position 1), 1/4 position(portion 2) and at the edge(position 3), respectively.

**Table 1:** Industrial trial scheme for IF steel.

Route No.	Route	Ca treatment
A	BOF-LF-RH-CC	Yes
B	BOF-RH-CC	No

**Table 2:** Average composition of steel (mass%) at the end of RH refining.

Type No.	C	Si	Mn	P	S	Als	Ti	Ca	Mg	T.O	N
A	0.005	0.010	0.12	0.010	0.01	0.045	0.06	0.0002	0.0007	0.0029	0.0044
B	0.003	0.018	0.15	0.008	0.004	0.038	0.06	0.003	0.001	0.0043	0.0045

**Table 3:** Average composition of slag at the end of RH refining, mass%.

Type	CaO	SiO_2_	MgO	Al_2_O_3_	Fe_2_O_3_	MnO	TiO_2_
A	53.18	8.05	5.74	20.36	1.69	0.44	0.3
B	39.79	13.53	8.10	26.86	3.45	1.34	0.94

**Figure 2:**
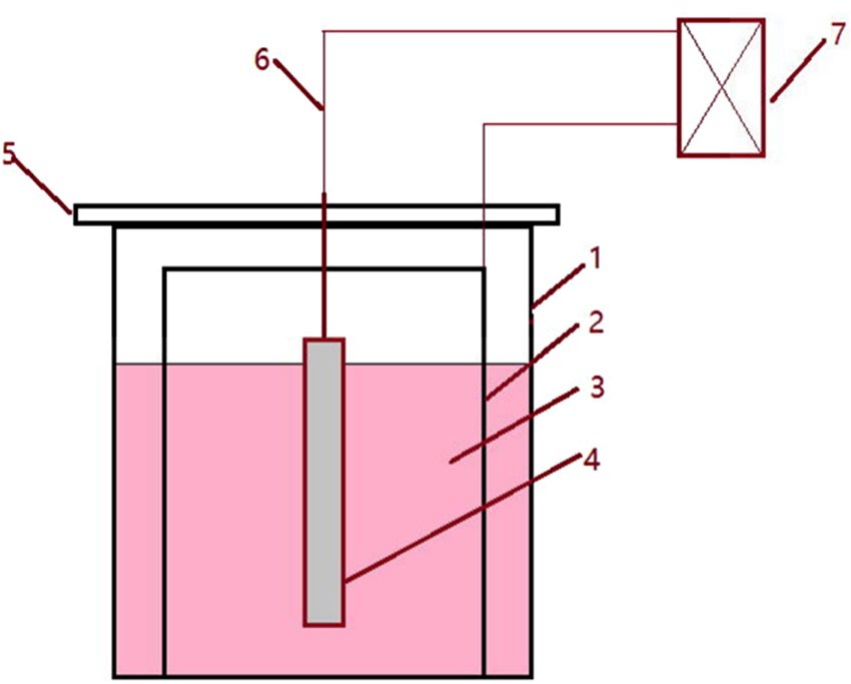
Schematic of apparatus for non-aqueous solution electrolysis (1- beaker, 2-stainless steel sheet, 3- solution, 4- specimen, 5- holder, 6-wire, 7-direct current power supply).

Two methods, i.e., traditional metallographic and electrolytic method, were applied to detect the non-metallic inclusion in a casting slab to obtain more information. For the first method, the steel samples were cut into 15 mm × 15 mm × 15 mm cubic specimens and then were ground and polished for SEM observation. Inclusions on the cross-section plane of each steel sample were detected and analyzed by SEM-EDS to obtain information of inclusions such as morphology, size and chemical compositions, etc. 20–30 inclusions per specimen were detected under 1000–5000 times magnification by this method. For the second method, the steel samples were cut into cylindrical shape with 10 mm in diameter and 80 mm in length. The apparatus for non-aqueous solution electrolysis is schematically shown in Fig. 2. The cylindrical specimen is used as anode and a stainless steel tube 50 mm in diameter and approximating 100 mm in length were applied as a cathode. The cell reactions occur on the anode and cathode are as reaction (1) and reaction (2), respectively. A type of methyl alcohol based electrolyte solution was developed to avoid the destruction of inclusion during electrolysis process, which containing 4–10 vol% glycerine, 4–10 vol% triethanolamine and 0.1–5 mass% tetramethylammonium chloride. During the experiment, the solution temperature was kept between 0–5 °C by putting the apparatus into a refrigerator, and the electric current density on the specimen surface dipped in the solution was maintained less than 200 mA/mm^2^. Generally, there are enough inclusion particles in the solution after the electrolyisis for 4 hours. Thereafter, the inclusions in the remaining solutions are separated and collected with the help of a centrifuge. Then the collected inclusions are dispersed by ultrasonic and this process repeated three times to remove the electrolyte completely. Finally, the collected inclusions are transferred on a conductive taps for SEM/EDS detection. In the present work, approximating 50 extracted inclusions per specimen were detected by EDS to determine their composition.

1 $${\rm{Fe}}={{\rm{Fe}}}^{{\rm{2}}+}+{{\rm{2e}}}^{-}$$

2 $$2{{\rm{CH}}}_{{\rm{3}}}{\rm{OH}}+{{\rm{2e}}}^{-}={{\rm{2CH}}}_{{\rm{3}}}{{\rm{O}}}^{-}+{{\rm{H}}}_{2(g)}\uparrow $$

**Figure 3:**
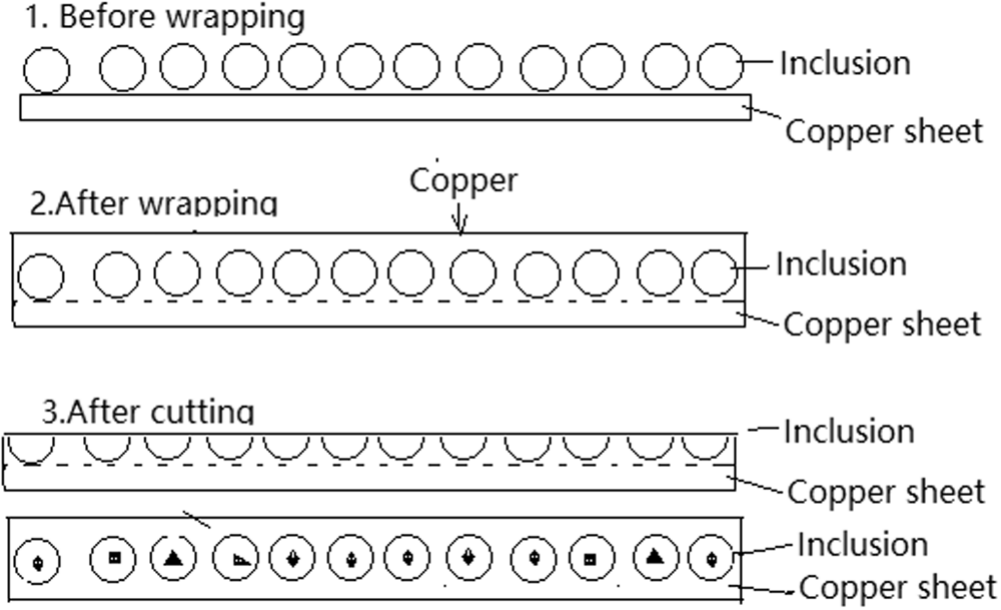
Schematic of wrapping and cutting the extracted inclusions by RTO technique.

In order to determine the inner structures of extracted inclusions, a RTO technique was also developed to wrap and cut the collected inclusions. First, the collected inclusions extracted by electrolysis were laid on a clean copper plate in a monolayer, which was then used as a cathode (another pure copper plate was used as an anode). The cell reactions on the anode and cathode for RTO technique are as reactions (3) and (4), respectively. The main steps of the RTO technique are schematically illustrated in Fig. 3. More details can be referred to our previous work^9^

3 $${\rm{Cu}}={{\rm{Cu}}}^{{\rm{2}}+}+{{\rm{2e}}}^{-}$$

4 $${{\rm{Cu}}}^{{\rm{2}}+}+{{\rm{2e}}}^{-}={\rm{Cu}}$$

## Results and Discussions

### Inclusions detected by traditional metallographic method

**Figure 4:**
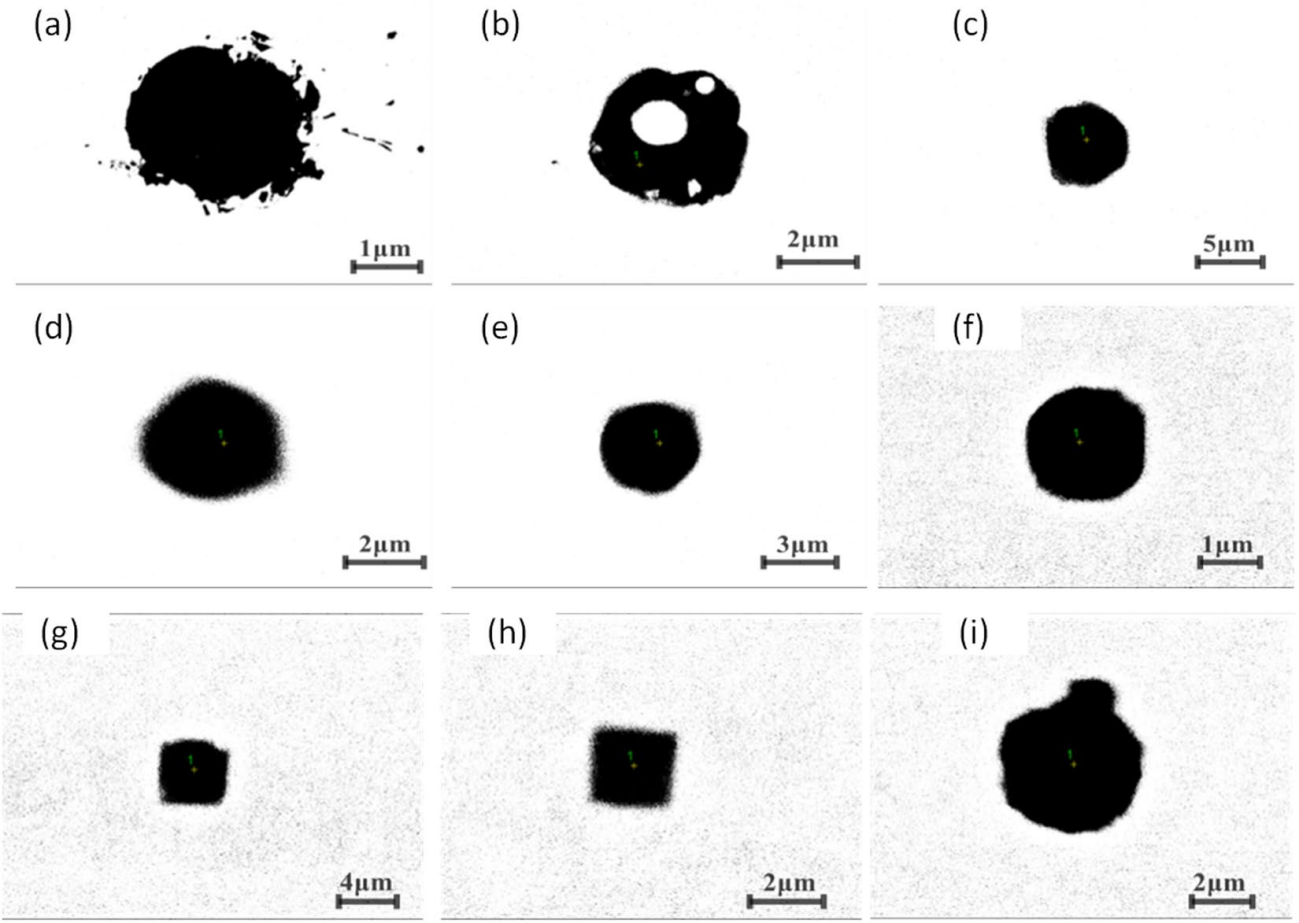
Morphologies of detected inclusions in different position of casting slab in route A.

**Figure 5:**
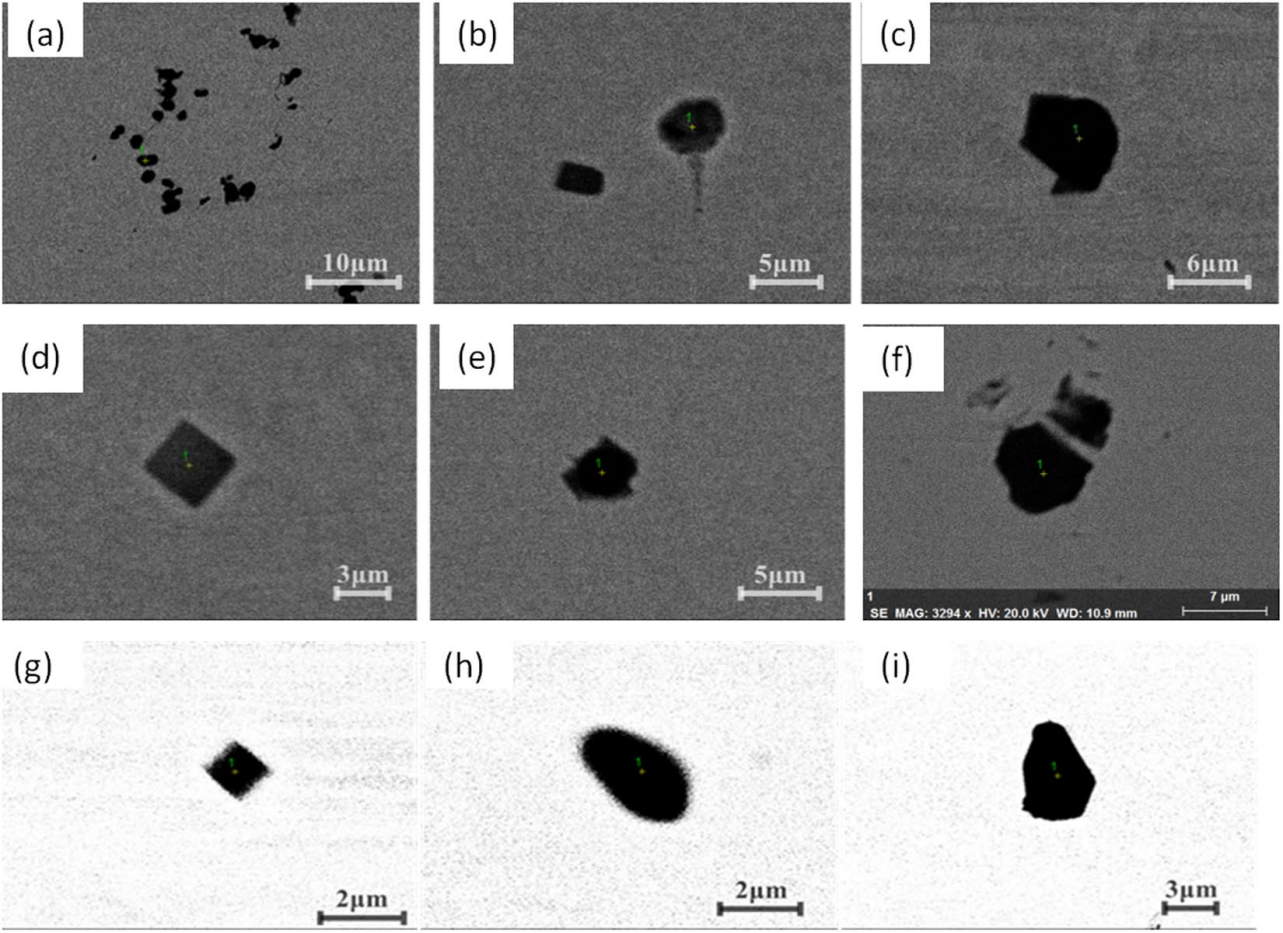
Morphologies of some typical inclusions in different position of casting slab in route B.

Figures 4 and 5 present the inclusions detected by traditional metallographic method of foregoing two different routes respectively. Figure 4 shows the typical inclusion morphology of specimens for route A. There are two main types of inclusion: the first type is spherical or spheroid in morphology those are main CaO-containing CaO-Al_2_O_3_-MgO based inclusion or Al_2_O_3_ based inclusion determined by EDS; and the other type is with a cubic morphology which is usually TiN type inclusion. The inclusion diameters are several micro-meters. Additionally, some complex duplex inclusions, i.e., TiN precipitating around oxide inclusion as a core, are also detected.

Figure 5 shows some typical inclusion morphologies in a slab for route B. There are also mainly two types of inclusions in the slab: oxides and nitrides with several micro-meters in size. But oxides are main Al_2_O_3_ based inclusion, generally with irregular morphology which are very different from those in route A as shown in Fig. 4. At the mean time, cubic-shaped TiN inclusions were also observed in the slab as a mainly inclusion type, which is similar to those of route A.

**Figure 6:**
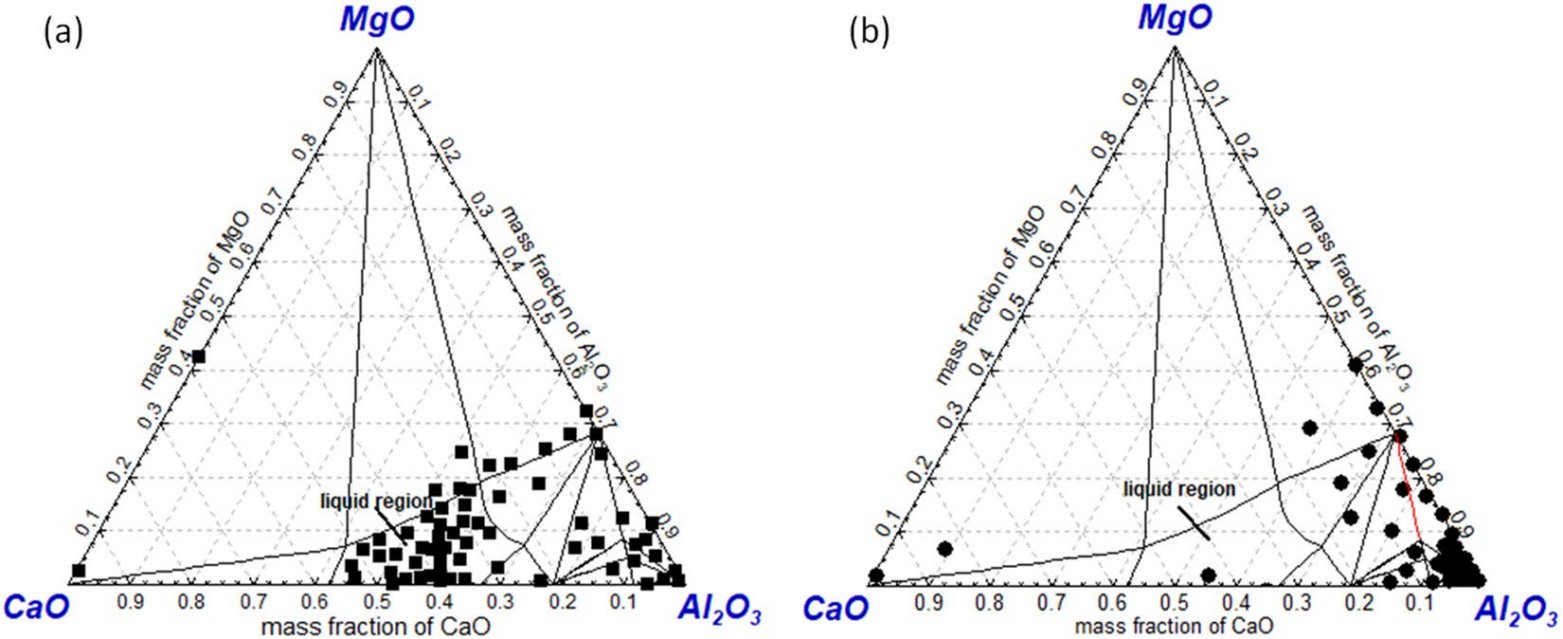
Composition of detected inclusions in a slab of route A(**a**) and route B(**b**) projected in CaO-Al_2_O_3_-MgO ternary phase diagram at 1873K (**a**) Route A, (**b**) route B

Figure 6(a,b) show the detected oxide inclusion composition of route A and route B projected in CaO-Al_2_O_3_-MgO ternary phase diagram at 1873K, respectively, as almost all inclusions are mainly composed of CaO, Al_2_O_3_ and MgO. It can be seen that majority of inclusions in route A are CaO-Al_2_O_3_ based inclusion locating in the liquid region in the phase diagram. Also, there are only a small amount of Al_2_O_3_ or MgAl_2_O_4_ spinel type inclusions as shown in Fig. 6(a). As a comparison, inclusions in route B are almost Al_2_O_3_ or MgAl_2_O_4_ spinel with high melting temperature as shown in Fig. 6(b). It should be pointed out very few CaO-rich inclusions were both detected in route A and route B. This is because a few number of CaO-SiO_2_ based inclusions were detected while no SiO_2_ content was projected in the phase diagrams.

In brief, the oxide inclusions in a slab of route A and route B show large differences from each other. In route A, majority of oxide inclusion have been modified into CaO-Al_2_O_3_-MgO based inclusion owing to Ca-treatment. However, oxides in route B are almost not modified at all due to no Ca-treatment and higher oxidizing refining slag. It should be pointed out that there are a large number of TiN precipitating in the slab of both routes since they have very similar Ti and N content.

### Inclusions detected by electrolysis methods and RTO technique

**Figure 7:**
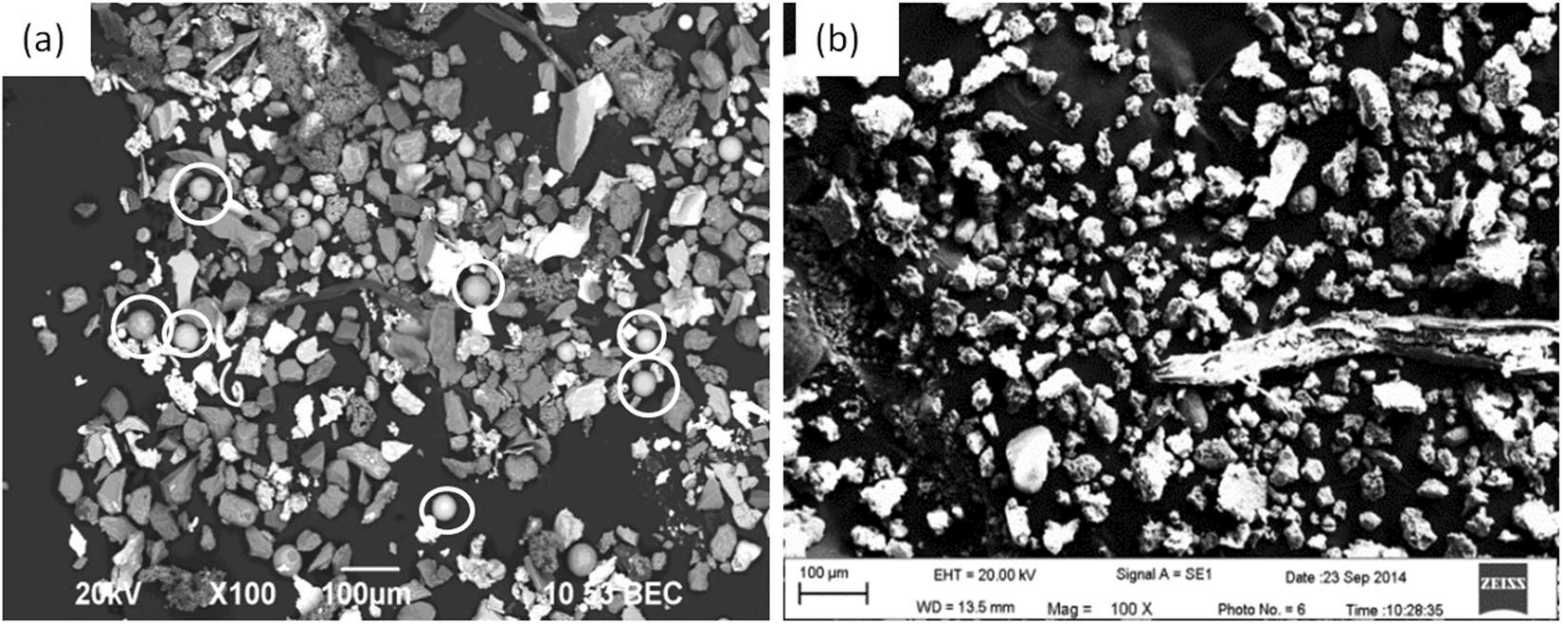
Morphologies of extracted inclusions by electrolysis method, (**a**) route A, (**b**) route B.

Table 4 shows the electrolysis specimen weight and extracted inclusion mass fraction in the foregoing both routes. The inclusion mass fraction in the three positions of a slab both in route A and route B almost do not show large different. The mass fraction of extracted inclusion in route A is much less than that in route B which is in agreement with the total oxygen listed in Table 2, while the extracted inclusion mass fraction seems less than that calculated by total oxygen suggesting some inclusion were perhaps lost during the collecting and transferring process after extraction. Figure 7 shows three dimensional morphologies of extracted inclusion for the foregoing two routes from position 1 by applying of electrolysis method. Inclusions in both routes do not appear so much difference like that detected by metallographic method. Inclusions in the both routes are mainly irregular in morphology and with a relative large size between several micro-meters to several hundred micro-meters. It should be stressed that a small number of spherical morphology inclusions can also be detected in extracted inclusion of route A as marked by white circles in Fig. 7(a).

**Table 4:** Extracted inclusion mass of specimens by electrolytic method.

Route		specimen weight/g	Specimen weight loss/g	Extracted inclusion weight/mg	Inclusion mass%
	Position 1	80.39	23.89	0.75	0.0032
**A**	Position 2	85.62	26.50	0.89	0.0034
	Position 3	84.78	23.80	0.81	0.0034
	Position 1	79.97	23.58	1.27	0.0054
**B**	Position 2	82.60	24.14	1.17	0.0051
	Position 3	83.06	24.98	1.22	0.0048

**Figure 8:**
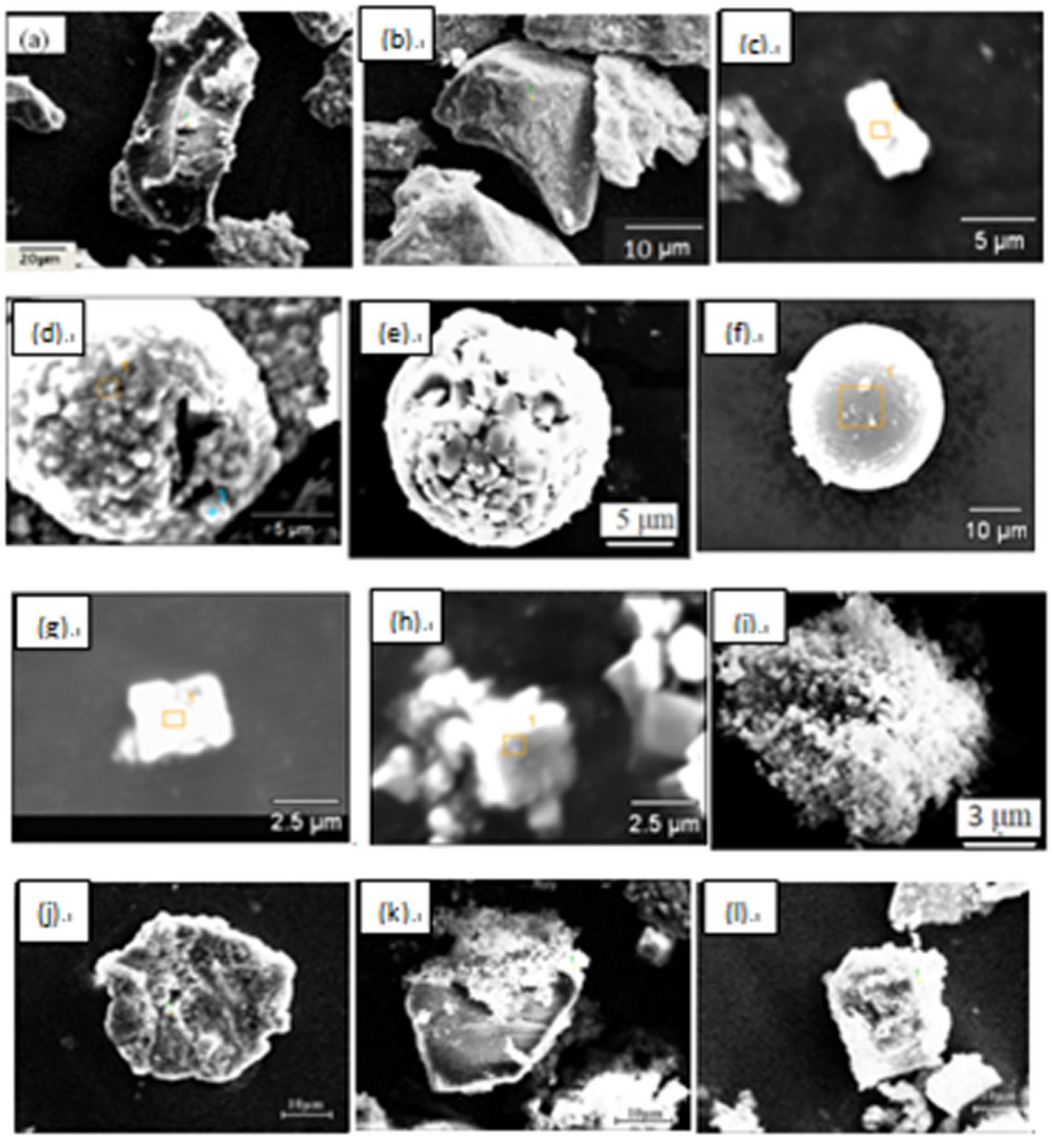
Some typical individual inclusion extracted by electrolysis method.

**Figure 9:**
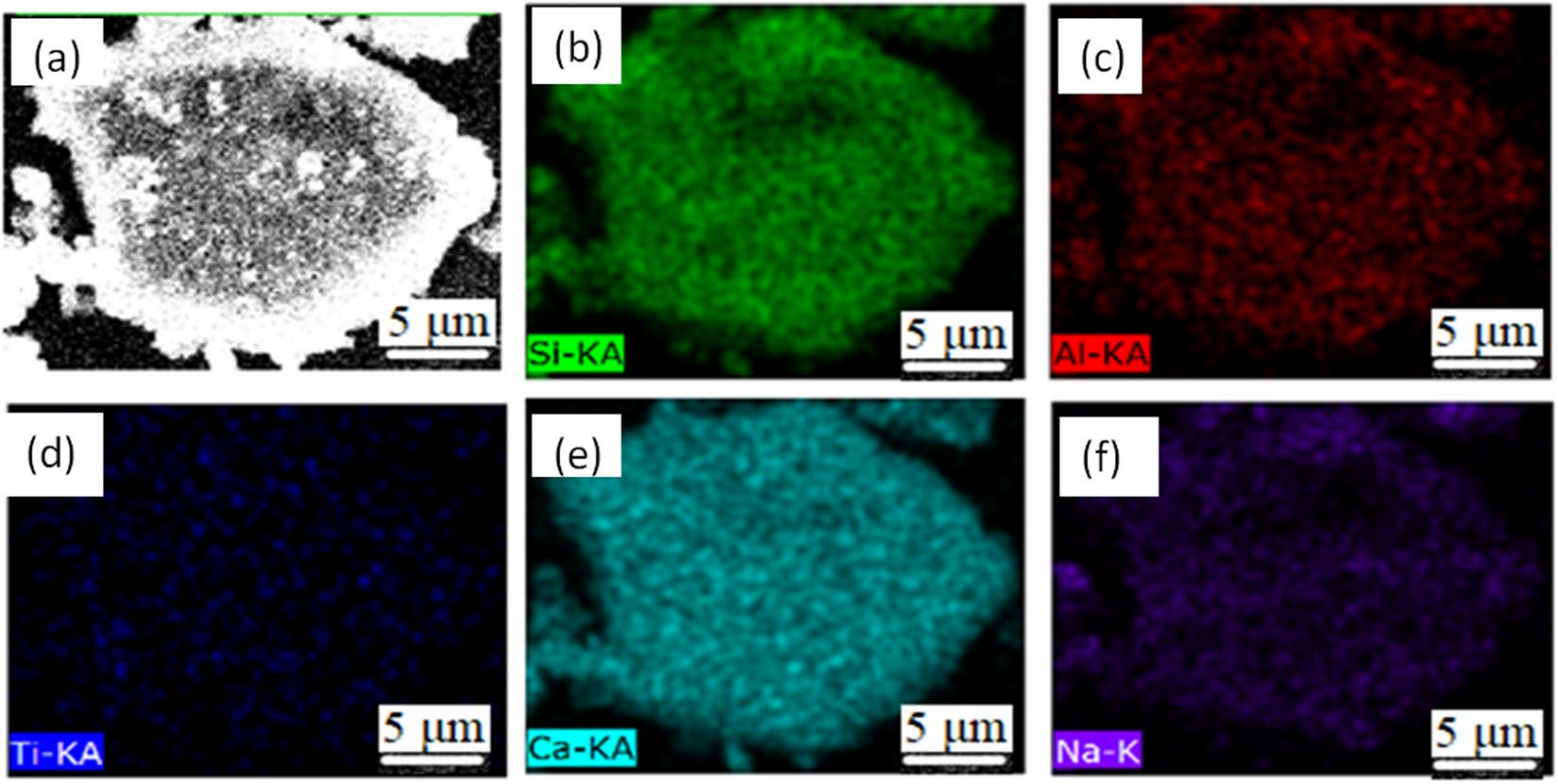
SEM-Mapping of a typical CaO-SiO_2_ based inclusion by electrolysis method.

Figure 8 shows some typical individual inclusions in both routes extracted by electrolysis methods correspondingly. Inclusions listed in Fig. 8(a–e) are Al_2_O_3_ based inclusion determined by EDS, in which Fig. 8(a,b) are Al_2_O_3_, Fig. 8(c) is MgOAl_2_O_3_ spinel, and Fig. 8(d,e) are typical aggregating Al_2_O_3_ based cluster. It should be pointed out that the above types of inclusion are detected both in route A and route B. Figure 8(f) shows a spherical CaO-Al_2_O_3_ based inclusion observed in route A and it was not detected in route B. Figure 8(g,h) present cubic TiN inclusions those are detected frequently in both route A and route B. Figure 8(i) displays a typical aggregating cluster that consists of many small titanium oxide particles which can also be detected in both routes. Figure 8(j–l) illustrate typical CaO-SiO_2_ based inclusions those are seldom detected by traditional metallographic method comparing to those inclusion morphology and composition presented in Figs 4–6. This type of CaO-SiO_2_ based inclusion usually has a irregular morphology and relative large size, majority of their size is more than 20 μm. Figure 9 shows SEM-mapping images of a typical CaO-SiO_2_ based inclusion, in which Ca and Si distribute almost homogenously and a small amount of Na is also detected within the inclusion. In addition, many small crystals are precipitated on the surface of the inclusion.

**Figure 10:**
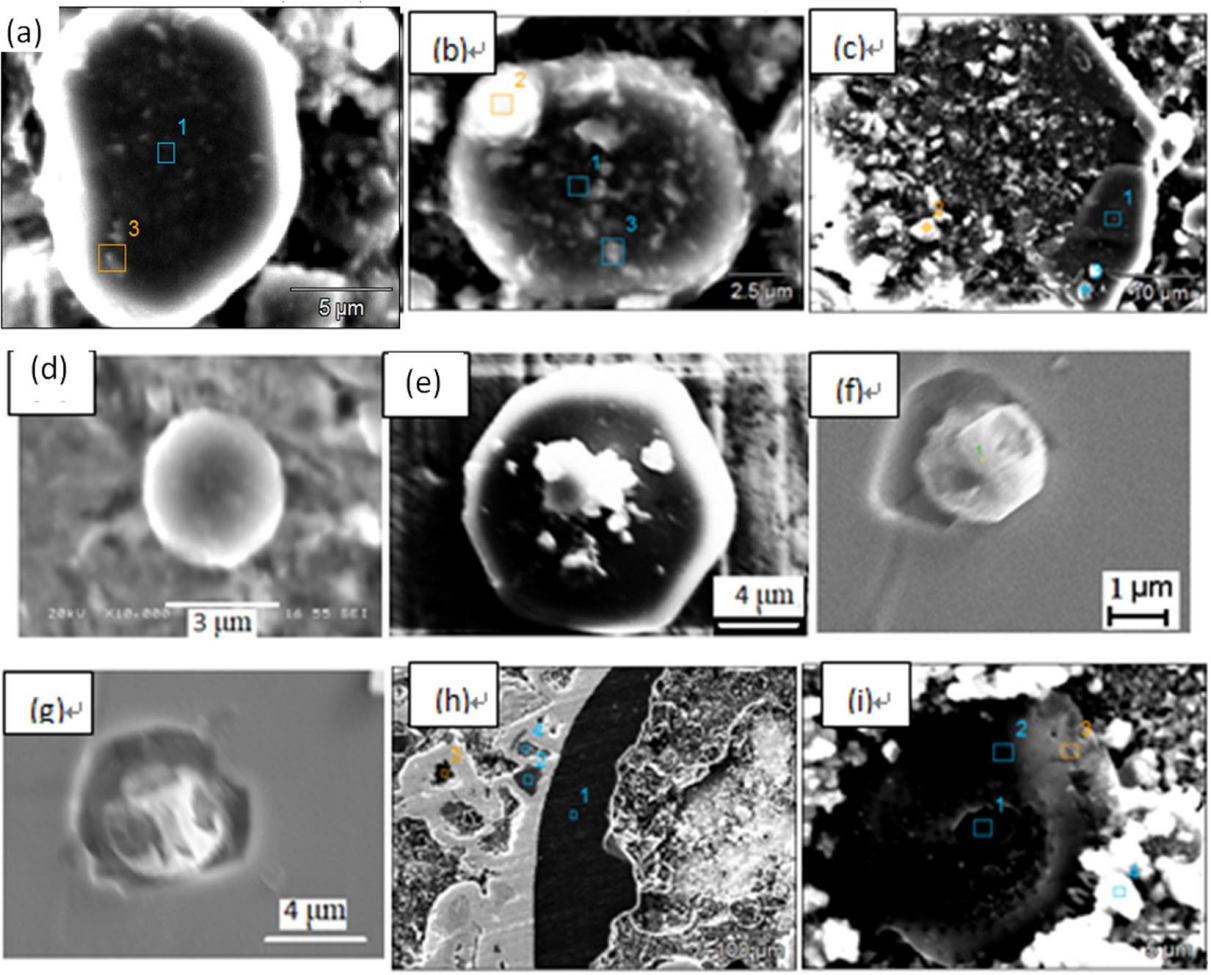
Inner structures of some typical extracted inclusion cut by RTO technique.

Figure 10 shows the inner morphology of some typical extracted inclusions from route A and route B, after being cut using our RTO technique. Figure 10(a,b) show the inner structures of individual Al_2_O_3_-based inclusions of route A and route B, respectively, which are very dense and no core observed by SEM within them. Figure 10(c,d) display two typical aggregating Al_2_O_3_-based inclusions from route A and route B respectively. They are composed of many small particles with similar inner structure though they are extracted from two different routes. Figure 10(e) shows a typical spherical CaO-Al_2_O_3_ based one from route B. Figure 10(f,g) present two TiN based inclusion from route A and B, respectively, in which TiN are clearly detected precipitating surrounding a oxide core. In addition, the inner core in Fig. 10(f) are CaO-Al_2_O_3_ based inclusion while the later one is Al_2_O_3_ based determined by EDS. Figure 10(h,i) present the inner morphology of the CaO-SiO_2_-based inclusion extracted from route A and from route B, respectively, in which no inner secondary dendrites precipitated within the inclusion, remaining as a glassy phase, while there are some small crystals precipitating on the edge or central part in the Fig. 10(i), in which CaO/SiO_2_ appear very large different in different part due to different crystallization.

**Figure 11:**
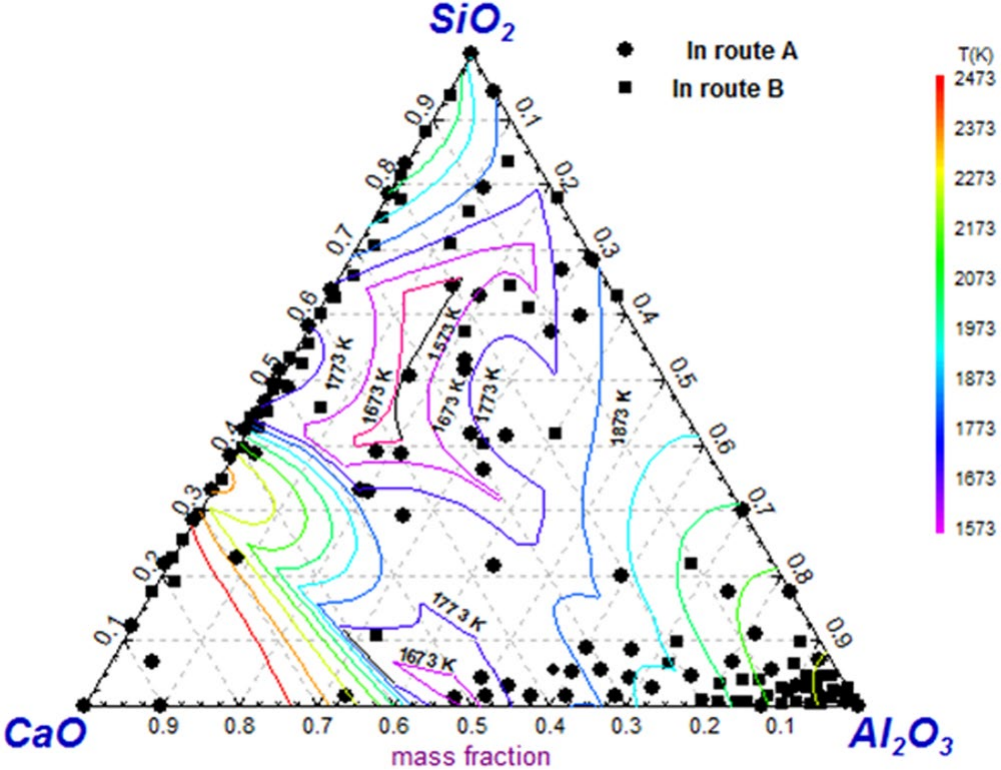
Inclusion composition by electrolysis method projected in SiO_2_-CaO-Al_2_O_3_ ternary iso-thermal phase diagram.

Figure 11 shows inclusion composition of both route A and route B detected by electrolysis method projected in SiO_2_-CaO-Al_2_O_3_ ternary phase diagram since majority of inclusions are mainly composed of CaO, Al_2_O_3_ and/or SiO_2_. The CaO-SiO_2_ based inclusions are much more compared with that presented in Fig. 5. It should be pointed out that CaO-SiO_2_ based inclusions present a huge composition variety scope in the phase diagram. The reason will be explained in the following paragraph

### Comparison of Inclusions by traditional metallographic method and electrolysis method

**Figure 12:**
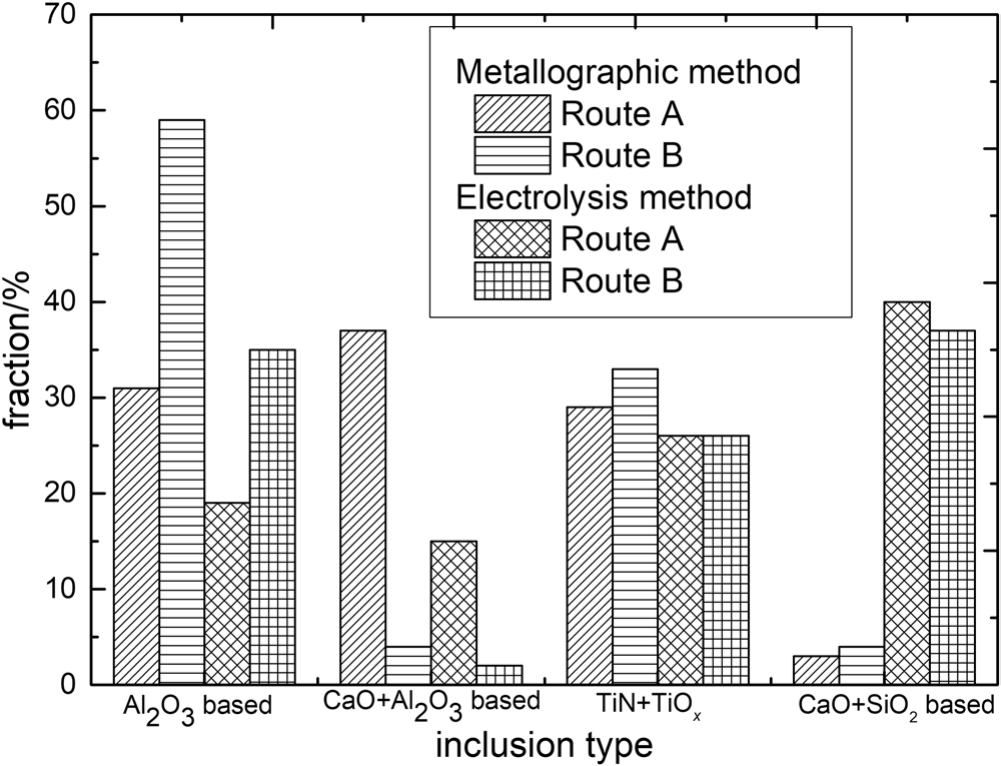
Frication of three types of typical inclusions by metallographic and electrolysis method.

Figure 12 shows fraction of main inclusion types in steel specimens of route A and route B. The detected inclusions by traditional metallographic method are mainly Al_2_O_3_ based, CaO-Al_2_O_3_ based, TiN or TiOx based. In contrast, a large number of CaO-SiO_2_ based inclusions up to approximating 40% were detected by applying of electrolysis method while it is nearly not observed by former method.

**Figure 13:**
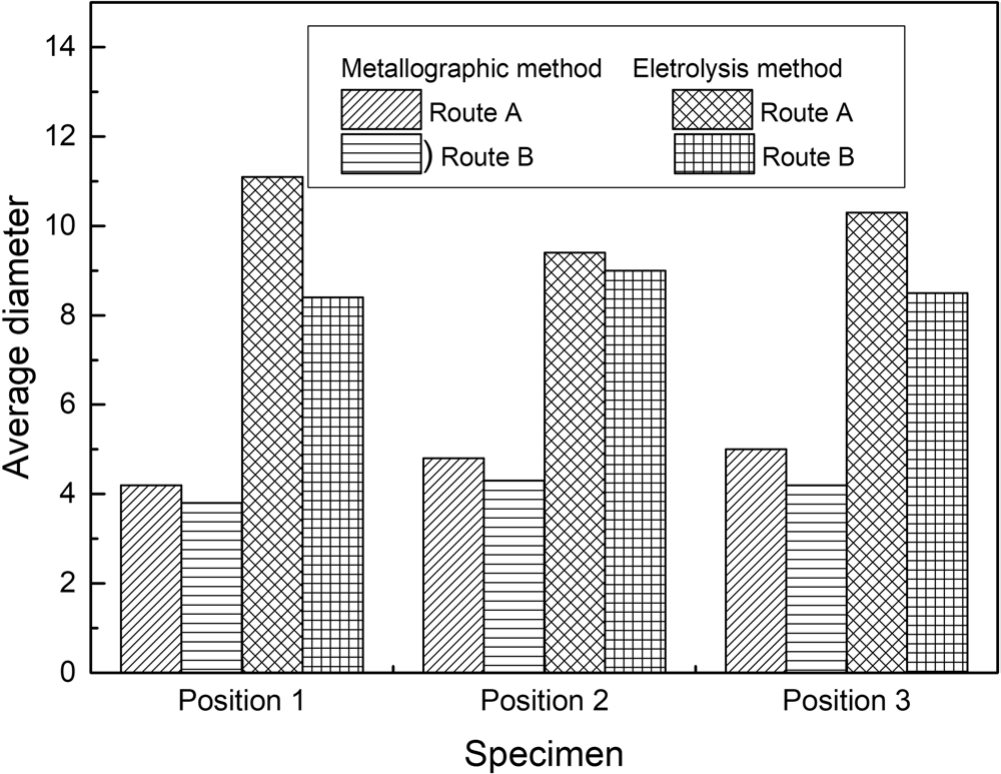
Average inclusion size at different position in a slab detected by metallographic and electrolysis method.

Figure 13 presents the average size evolution of inclusion in steel of route A and route B by two methods. It can be drawn that inclusions in the three positions do not display a specified tendency, while the inclusion size by electrolysis method appear obvious larger than that detected by metallographic method.

The reasons for the difference are that, one can usually detect the small size endogenous inclusions using a metallographic method since they have a larger number density. While it is easier to find out the large size inclusions by electrolysis method, since all the collected inclusions, including large size extraneous inclusion and small size endogenous inclusion, mixed together. In addition, some small size inclusions are perhaps missed during the collection process.

### Origin of SiO_2_-CaO based inclusion

Regarding Al_2_O_3_ based or TiN containing inclusion in ultra-low carbon IF steel, many researchers have reported thermodynamics for their precipitation^4–8^. Here we would not discuss them any longer since it has been well understood. The SiO_2_-CaO based inclusion, however, was not very well understood as this kind of inclusion is not likely to be an endogenous inclusion due to a very low Si content in liquid IF steel. It is very necessary to clarify the origin of SiO_2_-CaO based inclusion.

Compared to the composition of tundish flux and mold flux listed in the Table 5, CaO-SiO_2_ based inclusions containing some amount of alkaline oxides such as Na_2_O and K_2_O, shown as in Fig. 9, are usually considered from mold flux entrapment. However, many CaO-SiO_2_ based inclusions are detected free of alkaline oxide those are possible from tundish flux entrapment since they are very similar in composition. Also, this kind of CaO-SiO_2_ based inclusions have a possibility originating from mold flux entrapment since some alkaline oxides have relative low boiling temperature thus they are likely to be removed during the entrappment process due to the high temperature. Further, CaO-SiO_2_ based inclusion compositions are largely scattered on the diagram, as shown in. Figure 9. For example, CaO/SiO_2_ of CaO-SiO_2_ based inclusions fluctuate in a very huge region. The causes for this phenomena are perhaps as follows:(1) the CaO-SiO_2_ based inclusions might come from different slag or flux entrapment, such as mold flux and tundish flux, which have different CaO/SiO_2_ in composition; (2) the different degrees of chemical reactions between the liquid steel and entrapped slag/flux drops, such 4[Al] + 3(SiO_2_) = 2(Al_2_O_3_) + 3[Si], and/or [Ca] + (SiO_2_) = 2(CaO) + [Si], may cause the CaO/SiO_2_ of inclusion change; and 3) the entrapped extraneous inclusions would crystallize during the solidification process to precipitate crystal phases with different CaO/SiO_2_, such as 3CaO·SiO_2_, 2CaO·SiO_2_, 3CaO·2SiO_2_·CaF_2_, 2CaO·Al_2_O_3_·SiO_2_ and so on with higher CaO/SiO_2_, and glassy phase and some SiO_2_ based phase with relative low CaO/SiO_2_.

**Table 5:** Composition of tundish flux and mold flux in the industrial trial.

Composition	CaO	Al_2_O_3_	SiO_2_	MgO	F	Na_2_O	K_2_O	TiO_2_	CaO/SiO_2_
Tundish	25.96	35.91	16.70	4.54	—	0.19	0.24	6.24	1.55
Mold flux	39.63	3.01	31.75	1.48	8.64	11.92	0.65	0.17	1.24

As reported in Song *et al*.’s numerical simulating results^10^, the level fluctuation of CSP mold is much more than that in traditional plate caster mold due to its rapid casting speed, which suggests the possibilities for mold flux entrapment increase a lot. In addition, the tundish flux entrapment should be also paid attention since a large number of CaO-SiO_2_ based inclusions are detected very similar composition to those of tundish flux, which means they are most likely to originate from tundish flux entrapment.

## Conclusion

Metallographic, electrolytic method and RTO technique are applied to analyze the inclusion characteristics in an IF steel continuous casting slab. The morphology and composition of the inclusions obtained by different manners are significant different: the inclusions detected by metallographic method usually appear relative smaller size and are mainly Al_2_O_3_ based or TiN based in composition, whereas those extracted by electrolytic method usually have larger size and are much more calcium-silicate based. The three dimensional morphology and inner structures of non-metallic inclusion can be determined by combined with electrolysis method and RTO technique. It is very helpful for us to fully understand the formation and origin of inclusion in a continuous casting slab by combing these methods.

The large size calcium-silicate based inclusions are confirmed high possibilities originating from mold flux and tundish flux entrapment, which are less affected by the liquid steel composition; while the smaller ones are generally endogenous inclusion during the refining or solidification process which strongly depend on the liquid steel composition and temperature. The large size CaO-SiO_2_ based inclusions are verified the main cause of the surface defect in the rolled strip.
